# Predicting Cognitive Decline in Parkinson's Disease with Mild Cognitive Impairment: A One-Year Observational Study

**DOI:** 10.1155/2020/8983960

**Published:** 2020-10-28

**Authors:** Pei-Hao Chen, Fang-Yu Cheng, Shih-Jung Cheng, Jin-Siang Shaw

**Affiliations:** ^1^Graduate Institute of Mechanical and Electrical Engineering, National Taipei University of Technology, Taipei, Taiwan; ^2^Department of Neurology, MacKay Memorial Hospital, Taipei, Taiwan; ^3^Department of Medicine, MacKay Medical College, New Taipei City, Taiwan; ^4^Institute of Long-term Care, MacKay Medical College, New Taipei City, Taiwan; ^5^Department of Physical Therapy and Assistive Technology, National Yang-Ming University, Taipei, Taiwan; ^6^Institute of Mechatronic Engineering, National Taipei University of Technology, Taipei, Taiwan

## Abstract

We conducted an observational study to investigate clinical predictors of cognitive decline in patients with mild cognitive impairment (MCI), with a focus on patients with Parkinson's disease (PD) and Alzheimer's disease (AD). The study was performed with detailed neuropsychological testing, a portable device for gait analysis, and a comprehensive geriatric assessment for patients with MCI. Cognitive decline was defined as subjective cognitive impairment with an objective decline in the Mini-Mental State Examination (MMSE) ≥2 points at the one-year follow-up. Participants (*n* = 74) had a median age of 70 (interquartile range 60–79) years, and 45.9% of them were women. At the end of the study, 17.6% of the patients with MCI had a cognitive decline. Although no differences were observed between groups at the baseline cognitive study, patients with PD-MCI demonstrated more cognitive decline than patients with AD-MCI (28.6% vs. 7.7% *p* = 0.03). Patients with PD-MCI had more physical disabilities, including scores of instrumental activities of daily living (IADL), Tinetti balance, and gait scores, and some Timed Up and Go components. Initial Clinical Dementia Rating—Sum of Boxes score was a better predictor of future cognitive decline than MMSE in PD-MCI. For predicting the occurrence of cognitive decline in PD-MCI, the prediction accuracy increased from the reduced model (AUC = 0.822, *p* < 0.001) to the full model (a total of five independent variables, AUC = 0.974, *p* < 0.001). Given the potentially modifiable predictor, our findings also highlight the importance of identifying sleep quality and the ability to perform IADL.

## 1. Introduction

Several risk factors for the progression from mild cognitive impairment (MCI) to dementia have been identified, including age; female gender; lower educational attainment; an amnestic subtype of MCI; neuropsychiatric symptoms of anxiety, depression, or apathy; sleep disturbance; poor gait performance; obesity; metabolic syndrome; and common chronic medical conditions [1–5]. Parkinson's disease (PD) with MCI (PD-MCI) is an entity with a highly variable prognosis, including reversion to normal cognition, stability in the MCI state, or conversion to dementia [6]. Regardless of reversion to normal cognition or persistence, PD-MCI has a prognostic value for predicting dementia [7]. The pooled prevalence rate of PD-MCI in a meta-analysis was reported by 40% [8]. PD is known as a disease that presents gait disturbance before cognitive changes. A meta-analysis found that older adults with PD had weak or no association with incident dementia even for motor domains [9]. However, accumulated evidence supports that decreased gait velocity is associated with an elevated risk of cognitive decline and incident dementia in older adults without neurological overt disease [10]. The Framingham Offspring Cohort [11] showed that subjects with walking speeds of ≤1 m/s had a nearly three-fold increased risk of Alzheimer's disease (AD) (HR 2.92, *p*=0.019) for the entire study sample and an HR of 2.98 (*p*=0.020) for those aged ≥65 years. The most important clinical predictors of global cognitive decline in PD were neuropsychological tasks with a more posterior cortical basis, including semantic fluency and visuospatial construction at the baseline assessment [12]. The cognitive decline in PD involves complex interactions among a variety of factors, including motor and nonmotor symptoms, medical comorbidities, and psychosocial issues. Currently, there is a paucity of longitudinal evidence comparing the evolution of cognitive changes of MCI associated with the most common neurodegenerative disorders, namely, PD and AD. This study seeks to determine whether cognitive decline differs in PD-MCI and AD-MCI. We use data collected from a cohort study to investigate clinically available predictors of cognitive decline in patients with MCI, with a particular focus on PD-MCI.

## 2. Methods

### 2.1. Study Design

We conducted a single-center, noninterventional, prospective, observational cohort study of patients with MCI at the neurological department of the MacKay Memorial Hospital (Taiwan). We collected baseline and follow-up visit data over one year with at least three visits per participant. All participants were fully informed and provided their written, informed consent. The study was approved by the MacKay Memorial Hospital Institutional Review Board (18MMHIS005e and 18MMHIS152) and was conducted according to the standards set by the World Medical Association's Declaration of Helsinki.

### 2.2. Participants

**Figure 1:**
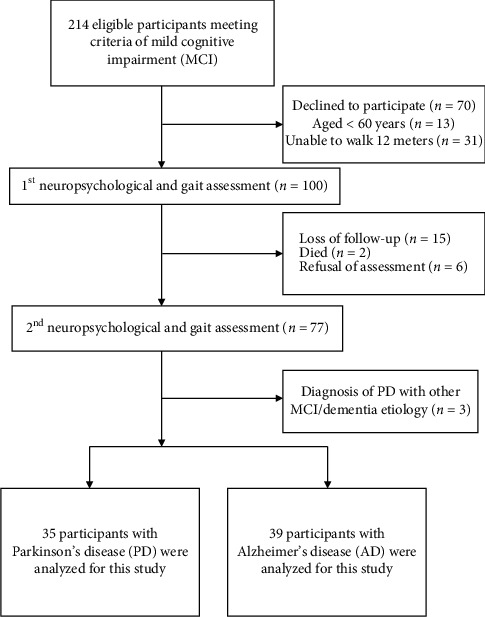
Flow chart of the selection of participants in this study.

A consecutive series of patients with MCI were recruited from April 2018 to July 2018. All participants met the following criteria: (a) age 60 years and older, (b) ability to walk 12 meters independently, (c) subjective cognitive complaint, and (d) no diagnosis of dementia. Participants were excluded if they had more than two incomplete test criteria within the tests for the MCI classification or uncontrolled medical conditions that cause walking abnormalities. A flow chart of participant selection is shown in Figure 1. All participants underwent a comprehensive neuropsychological evaluation by a trained nurse and were also examined by a board-certified neurologist. To avoid fatigue, the time limit for each interview was 90 minutes. The diagnosis of MCI was based on the global clinical dementia rating (CDR) of 0.5 and the CDR Sum of Boxes (CDR-SB) of 0.5–4.0 [13, 14].

### 2.3. Neuropsychological Testing and Geriatric Assessment

The study performed a comprehensive neuropsychological assessment of general and specific cognition, i.e., at least two tests per domain for each of the cognitive domains. These measures include the following: (1) global cognition (Mini-Mental State Examination [15] and CDR-SB [16]), (2) verbal learning and memory (California Verbal Language Test-II Short Form–immediate and delayed recall [17]), (3) processing speed/auditory working memory (Digits Recall Forward and Backward [18]), (4) semantic verbal fluency (animal naming [19]), (5) visuospatial processing and divided attention (Trail Making Test Parts A and B [20]), (6) visuoperceptual and visuospatial processing/memory (Benton Judgment of Line Orientation [21] and Taylor Complex Figure Test-Copy [22]), and (7) language (Boston Naming Test [23] and Cookie Theft Picture [24]). All participants underwent a geriatric comprehensive assessment with the Chinese version of the Geriatric Depression Scale-15 (GDS-15) [25], Pittsburgh Sleep Quality Index (PSQI) [26], Barthel Index [27], Lawton-Brody Instrumental Activities of Daily Living (IADL) [28], and Tinetti Gait and Balance Assessment Tool [29].

### 2.4. Wearable Gait Analysis

The straight-walking evaluation and Timed Up and Go (TUG) test were measured with the help of G-WALK® (BTS Bioengineering Corp

MA, United States), which is made up of a portable inertial measurement unit, the BTS® G-Sensor, precisely placed on L5 using an elastic belt. This hardware can acquire and transmit data to a PC through a Bluetooth connection. The software used is BTS® G-Studio (Copyright© BTS Bioengineering S. p. A.), which does data acquisition, elaboration, reporting, and storage. Data were collected using a sampling frequency of 100 Hz. The validity and reliability of movement performance have been well established [30]. Participants were asked to walk straight for 12 meters at a comfortable speed three times, and the average of the three trials was used for data analysis. In the TUG test, participants were instructed to stand up from a chair, walk 3 meters, turn around, walk back to the chair, and sit down three times. The total TUG duration and each time of the TUG component were recorded: time to stand, time to turn around in the midway, time to turn around to reach the chair, and time to sit down in the chair. The average of the three trials was used for data analysis. The BTS G-Studio has the protocols capable of analyzing the straight walking and TUG test, which automatically computes all the test parameters.

### 2.5. Defining of the MCI Etiologies and Follow-Up

The final diagnosis for the MCI etiology of each participant was reconfirmed by reviewing clinical data, neuropsychological data, brain imaging data, and biochemical tests at the end of the study. The diagnosis of PD was based on the Movement Disorder Society Task Force diagnostic criteria, either clinically established PD or clinically probable PD [31]. The clinical diagnosis of MCI due to AD was based on the National Institute on Aging and the Alzheimer Association workgroup consensus criteria [32]. MCI due to cerebrovascular disease, other rare etiologies (<3%), or at least two etiologies was excluded. Participants were followed for more than one year. Cognitive decline was defined as patient self-reported cognitive impairment with a decline of MMSE ≥ 2 points from baseline. The cutoff points were based on the regulation of the Taiwan National Health Insurance Administration [33]. Previous literature showed that the average intermediate progression rate is usually in the range of 2.0–4.9 points per year for AD [34] and 2.1–2.5 points per year for PD [35].

### 2.6. Statistical Analyses

Continuous variables with normal distribution were presented as mean and standard deviation; nonparametric or ordinal variables were reported as median and interquartile interval. Data normality and variance homogeneity were veriﬁed using the Shapiro–Wilk test and the Levene's test, respectively. We then performed univariate analyses to examine the differences in each continuous variable between groups using an independent *t*-test and the Mann–Whitney *U* test for variables with and without a normal distribution, respectively. Fisher exact tests were used for categorical variables. The level of significance was set at a*p* value of <0.05. Statistically significant variables in the analyses were included in the binary logistic regression model using the backward likelihood ratio (LR) method to identify factors associated with cognitive decline. We developed a reduced model for cognitive decline using the variables significant in the binary logistic regression analysis. All statistical analyses were performed using SPSS, version 25 (IBM, Armonk, NY, USA).

## 3. Results

### 3.1. Comparison of Demographics

A total of 214 people were initially identiﬁed, of whom 114 did not meet the inclusion criteria. The eligible 100 participants completed the first neuropsychological and gait assessment, and another 26 did not participate for a variety of reasons, including mixed etiologies, loss of follow-up, refusal of assessment, or having died. At the end of the study, 35 participants with PD and 39 with AD were analyzed for this study. Thus, the completion rate was *n* = 74 (74%). The flow diagram of eligible participants and enrolment into the study are listed in Figure 1.

Participants (*n* = 74) were aged between 60 and 90 years (median age 70 years, interquartile range 60–79), and 54.1% were male (*n* = 40). The median time interval between the onset of symptoms and the diagnosis was 2 years (interquartile range 1–3 years). The demographic and clinical characteristics of participants between PD-MCI and AD-MCI groups are shown in Table 1. Both the PD-MCI and the AD-MCI groups were similar regarding the age of visit, gender distribution, years of education, and body mass index. Analyses of cognitive data indicated that no significant differences were observed for global cognition and domain-specific cognition. Except for the basic gait parameters on straight walking, participants with PD-MCI showed poorer performance in some TUG components, including the time to stand and the time to turn around to reach the chair. Participants with PD-MCI had significantly more physical disabilities in IADL, Tinetti balance, and gait scores than did participants with AD-MCI. There was no significant difference in PSQI scores (6.65 vs. 6.39, *p*=0.771) and GDS-15 scores (3.24 vs. 2.69, *p*=0.479) between the groups.

After a one-year follow-up, four participants (2 AD-MCI and 2 PD-MCI) developed dementia, which was confirmed by the clinical criteria of final CDR ≥ 1 or CDR-SB ≥ 4.5 [13, 14]. Thirteen (17.6%) participants with MCI had a cognitive decline. Participants with PD-MCI demonstrated greater cognitive decline than those with AD-MCI (28.6% vs. 7.7%; *p*=0.03). Participants with PD-MCI who developed cognitive decline were significantly women more often than men, exhibited more severe IADL, had poorer sleep quality, and manifested worse performance across several neuropsychological tests, including CDR-SB, Forwards Digit Recall, and Boston Naming Test (Table 2).

The total time of TUG and its component, TUG End-turnings, have a marginal trend toward significance in predicting cognitive decline in PD-MCI (*p*=0.050 and *p*=0.051, respectively). There were no statistically significant differences in the parameters between cognitive decline and cognitive maintained groups in AD-MCI.

### 3.2. Logistic Regression Models

Based on the above ﬁndings, we further built a binary logistic regression model using the backward LR method and the signiﬁcant variables in Table 2 to predict the occurrence of cognitive decline in PD-MCI. Female gender, CDR-SB, Forwards Digit Recall, Boston Naming Test, and PSQI > 5 were included in the full model (Table 3). The significant independent predictors in the full model, female gender and CDR-SB, were then selected for analyses in the reduced model. For predicting the occurrence of cognitive decline, the prediction accuracy increased from the reduced model (AUC = 0.822, *p* < 0.001) to the full model (a total of five independent variables, AUC = 0.974, *p* < 0.001).

## 4. Discussion

To our knowledge, this study is the first to compare the longitudinal cognitive decline in PD-MCI and AD-MCI. The major finding of our research was that PD-MCI demonstrated more cognitive decline than AD-MCI after a one-year follow-up. Based on no significant differences in various baseline cognitive tests, and basic straight-line walking parameters between groups, patients with PD-MCI showed worse physical performance, including IADL, Tinetti Balance and Gait Assessment Tools, and some TUG components. A previous study showed baseline gait rather than cognitive status predicted specific cognitive decline in early PD [36]. In our study, participants with PD-MCI showed poor performance in some TUG components, including the time to stand and the time to turn around to reach the chair. We must emphasize that such activities are associated with the risk of falling [37] and future dementia [38]. Our study demonstrates the importance of adding gait assessments to cognitive testing on the prediction of cognitive decline.

Previous studies reported that specific items of the MMSE could provide early clues for quantifying the risk of future cognitive decline in MCI with good accuracy [39, 40]. In 2015, the Cochrane review did not support a substantial role of MMSE as a stand-alone, single-administration test in the identification of patients with MCI who could develop dementia [41]. The initial MMSE scores in our study were not significantly different between the groups with and without cognitive decline. Instead, initial CDR-SB was a better predictor of future cognitive decline than was MMSE in participants with PD-MCI. Since MMSE is purely a patient-based instrument, CDR-SB uses both the informant- and patient-based information, and a broader assessment of the cognitive and functional performance can be made.

Patients with PD-MCI that converted to dementia were older, and they had more deficits on the measures of attention, execution and verbal memory, sleep problems, smell dysfunction, and mood impairment at baseline [42–44]. In our study, participants with PD-MCI who developed cognitive decline had initially worse performance in Forwards Digit Recall and Boston Naming Test, which were used to assess processing speed/working memory and language function, respectively. The Boston Naming Test is never a single domain to assess language but gets affected by visuoperceptual skills (picture naming) [45]. Thus, both the frontal and posterior cortical dysfunction may define the etiology of cognitive decline in PD-MCI.

Among MCI due to AD, we found no association between the cognitive decline group and the cognitive maintained group, which was probably because only 3 participants had cognitive decline over a one-year follow-up.

Studies have suggested that sleep disturbances are associated with an increased risk of incident dementia and cognitive decline in the elderly and may be associated with psychological distress and depression [46]. A systematic review [47] that included five studies focusing on the risk factors for rapid cognitive decline in MCI, and higher IADL dependency, was associated with an increased risk of cognitive deterioration. In our study, two independent factors for cognitive decline in PD-MCI, IADL and PSQI > 5, became insignificant in the logistic regression model after adjusting for multiple confounders.

Several limitations must be addressed. First, the small sample size in the study may have limited the strength of the primary findings. This is attributable to the study involving a combination of detailed neuropsychological and gait assessment. Second, cognitive decline is inﬂuenced by a complex set of factors. There were still several known important factors, such as socioeconomic status, comorbid medical conditions, and complete medication information, which were not included in our study. Finally, all participants are still in the course of disease progression, and their cognitive status may change in the future. A more extended period of observational study involving other factors is ongoing. In conclusion, patients with PD-MCI exhibited a higher chance of cognitive decline than AD-MCI over a one-year follow-up. Initial CDR-SB was a better predictor of future cognitive decline in PD-MCI. Given the potentially modifiable predictor, our findings also highlight the importance of identifying sleep quality and the ability to perform IADL.

## Figures and Tables

**Table 1:** Comparison of demographic and clinical characteristics between PD-MCI and AD-MCI groups.

Group	PD-MCI *N* = 35	AD-MCI *N* = 39	*p* value
Cognitive decline (%)	10 (28.6)	3 (7.7)	0.030^*∗*^
Median age of visit	69.0	70.0	0.451
Male (%)	17 (48.6)	23 (59.0)	0.484
Education (year)	8.57	7.56	0.268
BMI	23.87	24.04	0.838
MMSE	25.80	24.92	0.285
CDR-SB	1.59	1.83	0.343
CVLT-SF immediate recall	5.47	5.46	0.985
CVLT-SF delayed recall	4.44	4.51	0.900
CVLT-SF recognition	5.56	5.13	0.470
TCF copy	31.26	30.00	0.445
TCF recall	13.98	13.76	0.912
JLO	13.70	13.37	0.703
Forwards digit recall	7.03	7.41	0.298
Backward digit recall	3.85	4.24	0.272
Trail making test A	30.08	23.33	0.150
Trail making test B	71.46	59.41	0.160
Verbal fluency animal	12.24	12.15	0.927
Boston naming Test	23.53	21.72	0.112
CTP Recall	13.13	14.08	0.516
CTP recognition	16.26	17.62	0.288
GDS-15 > 5 (%)	8 (24.2)	6 (15.4)	0.384
PSQI > 5 (%)	16 (51.6)	19 (48.7)	1.000
Barthel index	93.68	99.36	0.053
IADL	19.88	23.00	0.005^*∗*^
Tinetti balance	14.10	15.56	0.026^*∗*^
Tinetti gait	11.00	11.85	0.033^*∗*^
Walking speed (m/s)	0.822	0.878	0.267
Cadence (steps/min)	98.49	97.56	0.785
Stride length (cm)	66.06	67.72	0.765
TUG total (s)	19.97	15.72	0.080
TUG sit-to-stand (s)	1.94	1.68	0.034^*∗*^
TUG mid-turnings (s)	3.08	2.58	0.258
TUG end-turnings (s)	3.00	2.13	0.023^*∗*^
TUG stand-to-sit (s)	2.54	2.57	0.922

^*∗*^Statistically signiﬁcant *p* values compared between the groups (*p* < 0.05).

**Table 2:** Comparison of demographic and clinical characteristics between cognitive decline and cognitive maintained groups.

Group	PD-MCI cognitive decline group *N* = 10	PD-MCI cognitive maintained group *N* = 25	*p* value	AD-MCI *N* = 3	AD-MCI *N* = 36	*p* value
No (%)	10 (28.6)	25 (71.4)	—	3 (7.7)	36 (92.3)	—
Median age of visit	72	66	0.400	71	70	0.833
Male (%)	2 (20)	15 (60)	0.035^*∗*^	1 (33.3)	22 (61.1)	0.354
Education (year)	8.40	8.64	0.843	7.00	7.61	0.901
BMI	23.08	24.24	0.466	21.78	24.22	0.228
MMSE	26.50	25.52	0.339	25.33	24.89	0.709
CDR-SB	2.25	1.32	0.023^*∗*^	2.67	1.76	0.295
CVLT-SF immediate recall	5.00	5.67	0.148	4.00	5.58	0.140
CVLT-SF delayed recall	4.00	4.63	0.304	2.33	4.69	0.100
CVLT-SF recognition	5.20	5.71	0.445	3.00	5.31	0.112
TCF copy	30.06	31.75	0.254	28.00	30.17	0.100
TCF recall	11.22	15.11	0.219	5.00	14.49	0.126
JLO	12.67	14.08	0.462	13.33	13.37	0.799
Forwards digit recall	6.10	7.42	0.034^*∗*^	7.33	7.42	0.746
Backward digit recall	3.50	4.00	0.515	4.67	4.20	0.682
Trail making test A	26.89	31.28	0.953	21.67	23.48	0.759
Trail making test B	75.89	69.80	0.648	69.67	58.50	0.773
Verbal fluency animal	11.10	12.71	0.381	15.00	11.92	0.112
Boston naming Test	21.30	24.46	0.038^*∗*^	21.33	21.75	0.785
CTP recall	12.56	13.36	0.781	12.33	14.22	0.709
CTP recognition	16.22	16.27	0.915	16.33	17.72	0.599
GDS-15 > 5 (%)	4 (40.0)	4 (17.4)	0.170	3 (33.3)	5 (13.9)	0.376
PSQI > 5 (%)	8 (88.9)	8 (36.4)	0.009^*∗*^	3 (100)	16 (44.4)	0.068
Barthel index	91.00	94.79	0.539	100.00	99.31	0.823
IADL	16.90	21.13	0.042^*∗*^	22.33	23.06	0.295
Tinetti balance	13.00	14.62	0.348	15.67	15.56	0.823
Tinetti gait	9.90	11.52	0.105	12.00	11.83	0.823
Walking speed (m/s)	0.744	0.855	0.304	0.817	0.883	0.501
Cadence (steps/min)	101.77	97.13	0.642	90.44	98.17	0.294
Stride length (cm)	56.78	69.92	0.101	68.56	67.64	0.839
TUG total (s)	28.69	16.34	0.050	16.78	15.63	1.00
TUG sit-to-stand (s)	2.17	1.84	0.086	1.53	1.69	0.599
TUG mid-turnings (s)	4.06	2.65	0.081	2.34	2.60	0.941
TUG end-turnings (s)	4.20	2.48	0.051	1.90	2.14	0.785
TUG stand-to-sit (s)	2.97	2.37	0.079	2.17	2.60	0.374

*p* values calculated using fisher's exact test and Mann–Whitney *U* test, as appropriate. ^*∗*^Statistically significant result, *p* values <0.05.

**Table 3:** Binary logistic regression of predictors for cognitive decline in participants with PD-MCI.

Cognitive Decline				
Independent Variables	*B*	S. E.	Wald	*p* value

(Intercept)	0.315	6.074	0.003	0.959
Female	7.534	3.600	4.379	0.036^*∗*^
CDR-SB	3.502	1.681	4.342	0.037^*∗*^
Forwards digit recall	−0.938	0.703	1.782	0.182
Boston naming test	−0.154	0.278	0.306	0.580
PSQI > 5	−4.710	3.056	2.375	0.123

Multivariate classification results	Full model (all variables)		Reduced model (female + CDR-SB)	

Area under the curve	0.974		0.822	
*p* value	0.000^*∗*^		0.000^*∗*^	
Sensitivity	88.89%		40.00%	
Specificity	95.24%		92.00%	
Positive predictive value	88.89%		66.67%	
Negative predictive value	95.24%		79.31%	
Positive likelihood ratio	18.67		5	
Negative likelihood ratio	0.12		0.65	
Accuracy	93.33%		77.14%	

^*∗*^Statistically signiﬁcant *p*values compared between the groups(*p* < 0.05).

## Data Availability

The data used to support the findings of this study are available from the corresponding author upon request due to privacy/ethical restrictions.

## Abbreviations

AD: Alzheimer's disease
BMI: Body mass index
MCI: Mild cognitive impairment
MMSE: Mini-Mental State Examination
CDR-SB: Clinical Dementia Rating-Sum of Boxes
CVLT-SF: California Verbal Language Test-II Short Form
CTP: Cookie Theft Picture
GDS-15: Geriatric Depression Scale 15-Item
IADL: Instrumental Activities of Daily Living Scale
JLO: Judgment of Line Orientation
PD: Parkinson's disease
PSQI: Pittsburgh Sleep Quality Index
TCF: Taylor Complex Figure
TUG: Timed Up and Go.

## References

[1] Campbell N. L., Unverzagt F., LaMantia M. A., Khan B. A., Boustani M. A. (2013). Risk factors for the progression of mild cognitive impairment to dementia. *Clinics in Geriatric Medicine*.

[2] Assuncao N. (2018). Metabolic syndrome and cognitive decline in the elderly: a systematic review. *PLoS One*.

[3] Wennberg A. M. V., Wu M. N, Rosenberg P. B, Spira A. P (2017). Sleep disturbance, cognitive decline, and dementia: a review. *Seminars in Neurology*.

[4] Darweesh S. K. L., Licher S., Wolters F. J., Koudstaal P. J., Ikram M. K., Ikram M. A. (2019). Quantitative gait, cognitive decline, and incident dementia: the rotterdam Study. *Alzheimer’s & Dementia*.

[5] Vanoh D. (2019). Influence of gender disparity in predicting occurrence of successful aging, usual aging and mild cognitive impairment. *International Journal of Gerontology*.

[6] Fang C. (2020). Cognition deficits in parkinson’s disease: mechanisms and treatment. *Parkinson’s Disease*.

[7] Hoogland J., Boel J. A., De Bie R. M. A. (2017). Mild cognitive impairment as a risk factor for parkinson’s disease dementia. *Movement Disorders*.

[8] Baiano C., Barone P., Trojano L., Santangelo G. (2020). Prevalence and clinical aspects of mild cognitive impairment in parkinson’s disease: a meta-analysis. *Movement Disorders*.

[9] Kueper J. K., Speechley M., Lingum N. R., Montero-Odasso M. (2017). Motor function and incident dementia: a systematic review and meta-analysis. *Age and Ageing*.

[10] Quan M., Xun P., Chen C. (2017). Walking pace and the risk of cognitive decline and dementia in elderly populations: a meta-analysis of prospective cohort studies. *The Journals of Gerontology Series A: Biological Sciences and Medical Sciences*.

[11] Camargo E. C., Weinstein G., Beiser A. S. (2016). Association of physical function with clinical and subclinical brain disease: the Framingham offspring study. *Journal of Alzheimer’s Disease*.

[12] Williams-Gray C. H. (2007). Evolution of cognitive dysfunction in an incident parkinson’s disease cohort. *Brain*.

[13] O’Bryant S. E. (2010). Validation of the new interpretive guidelines for the clinical dementia rating scale sum of boxes score in the national alzheimer’s coordinating center database. *Archives of Neurology*.

[14] Wyman‐Chick K. A., Scott B. (2015). Development of clinical dementia rating scale cut‐off scores for patients with parkinson’s disease. *Movement Disorders Clinical Practice*.

[15] Folstein M. F., Folstein S. E., McHugh P. R. (1975). Mini-mental state. *Journal of Psychiatric Research*.

[16] Lynch C. A., Walsh C., Blanco A. (2006). The clinical dementia rating sum of box score in mild dementia. *Dementia and Geriatric Cognitive Disorders*.

[17] Delis D. C., Kramer J. H., Kaplan E., Ober B. A. (2000). *California Verbal Learning Test: Second Edition*.

[18] Blackwell (1961). *Distinctive Features of Learning in the Higher Animal. Brain Mechanisms and Learning*.

[19] Tombaugh T. N., Kozak J., Rees L. (1999). Normative data stratified by age and education for two measures of verbal fluency: FAS and animal naming. *Archives of Clinical Neuropsychology*.

[20] Lu L., Bigler E. D. (2000). Performance on original and a Chinese version of trail making test part B: a normative bilingual sample. *Applied Neuropsychology*.

[21] Benton A. L. (1983). *Judgment of Line Orientation*.

[22] Taylor L. B. (1969). Localisation of cerebral lesions by psychological testing. *Neurosurgery*.

[23] Chen T.-B., Lin C.-Y., Lin K.-N. (2014). Culture qualitatively but not quantitatively influences performance in the Boston naming test in a chinese-speaking population. *Dementia and Geriatric Cognitive Disorders Extra*.

[24] Goodglass H., Kaplan E., Barressi B. (1983). *Cookie Theft Picture: Boston Diagnostic Aphasia Examination*.

[25] Liu C. Y., Wang S. J., Teng E. L. (1997). Depressive disorders among older residents in a Chinese rural community. *Psychological Medicine*.

[26] Buysse D. J., Reynolds C. F., Monk T. H., Berman S. R., Kupfer D. J. (1989). The Pittsburgh sleep quality Index: a new instrument for psychiatric practice and research. *Psychiatry Research*.

[27] Mahoney F. I., Barthel D. W. (1965). Functional evaluation: the barthel index. *Maryland State Medical Journal*.

[28] Lawton M. P., Brody E. M. (1969). Assessment of older people: self-maintaining and instrumental activities of daily living. *The Gerontologist*.

[29] Tinetti M. E. (1986). Performance-oriented assessment of mobility problems in elderly patients. *Journal of the American Geriatrics Society*.

[30] Kleiner A. F. R., Pacifici I., Vagnini A. (2018). Timed up and go evaluation with wearable devices: validation in parkinson’s disease. *Journal of Bodywork and Movement Therapies*.

[31] Postuma R. B., Berg D., Stern M. (2015). MDS clinical diagnostic criteria for parkinson’s disease. *Movement Disorders*.

[32] Albert M. S. (2011). The diagnosis of mild cognitive impairment due to alzheimer’s disease: recommendations from the national institute on aging-alzheimer’s association workgroups on diagnostic guidelines for alzheimer’s disease.. *The Journal of the Alzheimer’s Association*.

[33] Yang Y. (2016). Dementia in Taiwan area. *Translational Neuroscience and Clinics*.

[34] Doody R. S., Massman P., Dunn J. K. (2001). A method for estimating progression rates in alzheimer disease. *Archives of Neurology*.

[35] Aarsland D. (2004). The rate of cognitive decline in parkinson disease. *Archives of Neurology*.

[36] Morris R., Lord S., Lawson R. A. (2017). Gait rather than cognition predicts decline in specific cognitive domains in early parkinson’s disease. *The Journals of Gerontology: Series A*.

[37] Nocera J. R., Stegemöller E. L., Malaty I. A., Okun M. S., Marsiske M., Hass C. J. (2013). Using the timed up & go test in a clinical setting to predict falling in parkinson’s disease. *Archives of Physical Medicine and Rehabilitation*.

[38] Lee J. E., Shin D. W., Jeong S.-M. (2018). Association between timed up and go test and future dementia onset. *The Journals of Gerontology: Series A*.

[39] Guerrero-Berroa E., Luo X., Schmeidler J. (2009). The MMSE orientation for time domain is a strong predictor of subsequent cognitive decline in the elderly. *International Journal of Geriatric Psychiatry*.

[40] Xie H., Mayo N., Koski L. (2011). Predictors of future cognitive decline in persons with mild cognitive impairment. *Dementia and Geriatric Cognitive Disorders*.

[41] Arevalo-Rodriguez I. (2015). Mini-mental state examination (MMSE) for the detection of alzheimer’s disease and other dementias in people with mild cognitive impairment (MCI). *Cochrane Database of Systematic Reviews*.

[42] Tsiouris K. M. (2019). A risk stratification model for early cognitive impairment after diagnosis of parkinson’s disease. *Mediterranean Conference on Medical and Biological Engineering and Computing*.

[43] Pedersen K. F., Larsen J. P., Tysnes O.-B., Alves G. (2013). Prognosis of mild cognitive impairment in early parkinson disease. *JAMA Neurology*.

[44] Chung S. J., Park Y. H., Yun H. J. (2019). Clinical relevance of amnestic versus non-amnestic mild cognitive impairment subtyping in parkinson’s disease. *European Journal of Neurology*.

[45] Yochim B. P., Kane K. D., Mueller A. E. (2009). Naming test of the neuropsychological assessment battery: convergent and discriminant validity. *Archives of Clinical Neuropsychology*.

[46] Guarnieri B., Sorbi S. (2015). Sleep and cognitive decline: a strong bidirectional relationship. It is time for specific recommendations on routine assessment and the management of sleep disorders in patients with mild cognitive impairment and dementia. *European Neurology*.

[47] Song Y.-N., Wang P., Xu W. (2018). Risk factors of rapid cognitive decline in alzheimer’s disease and mild cognitive impairment: a systematic review and meta-analysis. *Journal of Alzheimer’s Disease*.

